# Some reef-building corals only disperse metres per generation

**DOI:** 10.1098/rspb.2023.1988

**Published:** 2024-07-24

**Authors:** Katharine E. Prata, Pim Bongaerts, John M. Dwyer, Hisatake Ishida, Samantha M. Howitt, James P. Hereward, Eric D. Crandall, Cynthia Riginos

**Affiliations:** ^1^ School of the Environment, The University of Queensland, Saint Lucia, Queensland, Australia; ^2^ California Academy of Sciences, San Francisco, CA, USA; ^3^ The Caribbean Research and Management of Biodiversity (CARMABI) Foundation, Willemstad, Curaçao; ^4^ School of Chemistry and Molecular Biosciences, The University of Queensland, Saint Lucia, Queensland, Australia; ^5^ Department of Biology, Pennsylvania State University, University Park, PA, United States

**Keywords:** dispersal distance, Scleractinia, population genomics, isolation by distance, photogrammetry, cryptic species

## Abstract

Understanding the dispersal potential of different species is essential for predicting recovery trajectories following local disturbances and the potential for adaptive loci to spread to populations facing extreme environmental changes. However, dispersal distances have been notoriously difficult to estimate for scleractinian corals, where sexually (as gametes or larvae) or asexually (as fragments or larvae) derived propagules disperse through vast oceans. Here, we demonstrate that generational dispersal distances for sexually produced propagules can be indirectly inferred for corals using individual-based isolation-by-distance (IbD) analyses by combining reduced-representation genomic sequencing with photogrammetric spatial mapping. Colonies from the genus *Agaricia* were densely sampled across plots at four locations and three depths in Curaçao. Seven cryptic taxa were found among the three nominal species (*Agaricia agaricites*, *Agaricia humilis* and *Agaricia lamarcki*), with four taxa showing generational dispersal distances within metres (two taxa within *A. agaricites* and two within *A. humilis*). However, no signals of IbD were found in *A. lamarcki* taxa and thus these taxa probably disperse relatively longer distances. The short distances estimated here imply that *A. agaricites* and *A. humilis* populations are reliant on highly localized replenishment and demonstrate the need to estimate dispersal distances quantitatively for more coral species.

## 1. Introduction

The dispersal capacity of species determines their ability to recover from disturbances [1,2], expand their ranges [3], track ancestral habitat under changing environments [4] and spread adaptive alleles across the landscape [5]. Given globally pervasive environmental changes and habitat alterations, estimating species’ dispersal capacities would improve predictions for possible ecological and evolutionary future trajectories [6]. For these reasons, understanding dispersal dynamics in coral reefs has become a critical goal. Hard corals are integral to the physical and trophic structure of coral reef ecosystems yet are declining at unprecedented rates owing to local and global impacts, in particular, bleaching and mortality induced by marine heat waves [7]. Clarifying dispersal distances and their rates for coral species can therefore provide insights into reef recovery processes and adaptation to changing ocean conditions. For instance, short-distance dispersers may be quicker to repopulate following disturbances (from local sources), whereas recovery from large-scale disturbances and adaptive gene flow (or range shifts) would be more likely facilitated by long-distance dispersal [8]. However, little is known about dispersal distances within corals.

Physically tracking the dispersal of microscopic coral larvae is impractical; however, population genetic models allow dispersal to be inferred either directly or indirectly [9,10]. Direct measurements of sexually derived larval dispersal distances for corals are based on assigning a young colony back to its population or parent of origin. Population assignment methods have been primarily employed to give rough estimates of direct dispersal (e.g. [11,12]), but these methods require differences in allele frequencies between populations (i.e. population structure measured as *F*_*ST*_) to be relevant to recent timescales [13] and that all possible source populations are sampled [14]. Parentage analysis instead uses Mendelian expectations to assign a young colony to a parent colony and has been used to estimate direct dispersal distances in the fire coral, *Millepora* cf. *platyphylla*, where most offspring settled within a few hundred metres [15]. However, this method requires extensive genotyping and is therefore infeasible for species that are either highly abundant or that disperse longer distances. Furthermore, direct estimates are only relevant to one generation and thus may not represent time-average patterns (direct estimates could be higher or lower than average with downward bias more likely as dispersal events outside the limits to sampling are not captured). Thus, direct methods are usually unsuitable for estimating dispersal distance averages across multiple species with varying dispersal strategies.

Indirect methods rely on various population genetic models, with gene flow estimates being used as a proxy for larval dispersal. Most indirect studies of dispersal thus far have focused on describing population structure (*F*_*ST*_) under the heavily strained assumptions of the island model [16] and can be skewed by the presence of undetected cryptic taxa (i.e. causing upwardly or downwardly biased structure patterns at different spatial scales [17]). Despite these issues, most coral population structure studies support the expectation that sexual reproductive mode (brooder versus broadcaster) predicts relative dispersal, where brooders show population genetic structure across smaller spatial scales than broadcasters (e.g. [18]). This pattern has long been suspected from a shorter pelagic larval duration in brooders relative to broadcast spawners (hours to days versus days to weeks) [19].

Because the island model assumes equal gene flow among all modelled populations, it cannot be used to estimate dispersal distance. A population genetic model alternative to the island model is the isolation-by-distance (IbD) model, which uses the expected increase in genetic distance (or the decrease in relatedness) with increasing geographic distance [20–22]. IbD methods require less intensive sampling than parentage and can yield statistics describing generational dispersal distances (*σ*) as recent multigenerational averages when paired with information on individual density [23,24]. However, IbD methods assume a migration-drift equilibrium across the spatial scale being analysed [25,26]. Through evidence by simulations, spatial scales from *σ* to 10*σ*−50*σ* are most likely within migration-drift equilibrium [27,28] and, therefore, represent the ideal distances for analysis. The IbD model has been used extensively to measure dispersal distances in plants (e.g. [28]) and shows great promise for marine species with the IbD-derived estimates from marine fishes closely aligning with direct estimates [29,30].

While IbD correlations are commonly employed in coral studies, dispersal distances are rarely estimated, probably because of the difficulties in accurately measuring individual density, a crucial parameter for estimating dispersal distances that is difficult to resolve. To date, only three species of coral have had dispersal distances estimated using the IbD method. The brooding octocorallian *Corallium rubrum* had generational dispersal distances across centimetres [31,32], the scleractinian *Pocillopora damicornis* (in Hawaii, where it broods [33]) had a dispersal distance of a few metres [34] and the scleractinian broadcaster *Acropora palmata* had a dispersal distance of 1 km using population-level metrics [35]. To estimate dispersal distance, all three studies relied on census *N* for individual density calculations (with extrapolation for *A. palmata*) rather than more appropriate individual genetic effective densities (using *N_e_*). Additionally, none of these studies used modern genomic methods which are far more powerful in resolving cryptic taxa and detecting subtle spatial signatures.

For scleractinian corals, asexually derived dispersal is better understood than sexually derived dispersal, with distances and rates of asexual dispersal varying substantially. Asexual reproduction can be rampant in certain species, with coral colonies of only a few genotypes covering large spatial areas [34,36]. Besides dispersal through fragmentation, some corals produce larvae parthenogenically [37] and these larvae may be able to travel distances equivalent to sexually derived larvae. Indeed, parthenogenic asexual dispersal can be a highly informative way to characterize larval dispersal distances [38], although like parentage asessments it is likely to miss long distance dispersal events. Thus, the distribution of genotypes can be heavily influenced by asexual dispersal and measuring the clonal propensities of different coral species, along with sexually derived dispersal, provides a more complete picture of coral dispersal.

To simultaneously estimate sexual and asexual dispersal distances, especially over short spatial scales, the location of each coral colony needs to be mapped precisely. Few studies have tracked individual colony positions and estimated their genetic relationships (notable exceptions include [15,32,34,39]). Precise mapping can be achieved by implementing structure-from-motion photogrammetry which uses imagery to create fine-scale 3D models of reef structure (i.e. [40]), but has not yet been used to estimate dispersal.

In the present study, spatial positions inferred by photogrammetry are connected to individual genomic profiles to robustly characterize dispersal for multiple co-distributed coral taxa using IbD methods. We estimate dispersal distances for populations in the scleractinian coral genus *Agaricia*—a dominant member of reefs throughout the Caribbean. *Agaricia* have been described as having ‘weedy’ life-histories [19,41], because they brood their larvae (although reproductive mode has only been assessed in 3 out of 7 species, namely, *Agaricia agaricites*, *Agaricia humilis* and *Agaricia tenufolia* [42,43]), colonize disturbed areas [41] and have increased in relative abundance in the Caribbean against a backdrop of overall decreasing hard coral [44]. Despite this weedy reputation, this genus is also declining in absolute abundance [45] and is an important element of Caribbean coral reef biodiversity. For instance, *Agaricia* is one of the more speciose genera in the Caribbean [46], covers substantial reef area (e.g. 41% of coral cover between 10 m and 40 m depth in Curaçao and Bonaire [44]), can be found from the shallowest to the deepest depths for photosynthetic organismal limits [47] and often dominates mesophotic reefs [48]. Morphologically cryptic but genetically divergent groups have been found within *Agaricia fragilis*, *Agaricia lamarcki* and *Agaricia grahamae*, with segregation by depth noted for *A*. *fragilis* and *A*. *lamarcki* [49,50].

Taking advantage of this diverse assemblage of co-occurring taxa, we measure dispersal distances across the same spatial areas and explicitly test whether dispersal distances are similar among congeners. We also test whether dispersal differs between vertical (depth) and horizontal distance by using a depth-replicated sampling design. We demonstrate that both sexual and asexual dispersal distances can be very short in corals (within metres for *A. humilis* and *A. agaricites*) and incongruent among congeners, where *A. lamarcki* likely disperse further. This study presents a novel application of structure-from-motion photogrammetry for spatially explicit population genomic studies on benthic organisms such that dispersal distance can be compared for multiple coral species replicated across multiple locations.

## 2. Material and methods

### (a) Study sites, sample collection and structure-from-motion photogrammetry

**Figure 1.**
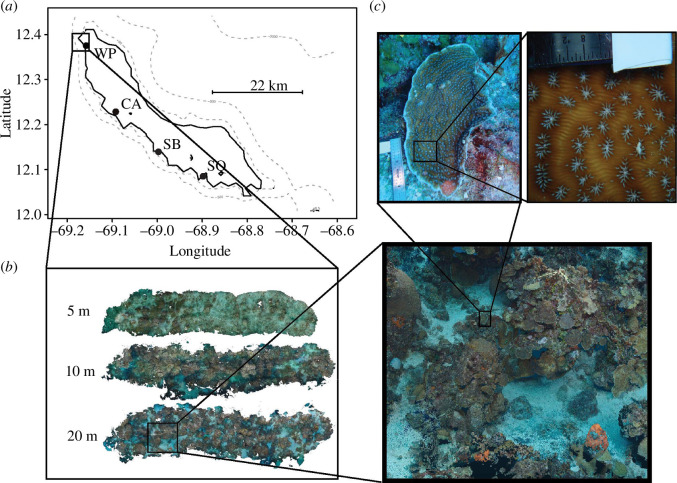
Locations and sampling design for spatial genetic analysis and dispersal distance estimation of *Agaricia spp.* (*a*) Map of Curaçao (Southern Caribbean) and the four sampling locations (WP: West Point ‘Playa Kalki’, CA: Cas Abao, SB: Snake Bay and SQ: Seaquarium). (*b*) Within each location, three (two within SQ) photogrammetry plots were imaged with dimensions of 25 m length by 4 m width*.* (*c*) Dense sampling of colonies of *Agaricia spp*. was conducted within plots (at 25 × 2 m) where all colonies were photographed and tissue sampled for genomic sequencing (RADseq).

*Agaricia* spp. samples were collected in 2019 on the leeward side of Curaçao (figure 1) from permanent plots (4 × 25 m with a long axis maintaining constant depth) from the CoralScape long-term monitoring project [40]. Locations were approximately 10 km apart (West Point, Cas Abao, Snake Bay and Seaquarium), and adjacent plots were made at three depths (5, 10 and 20 m) within each location, except at Seaquarium, where plots were at two depths (12 and 20 m) because of the initial steepness of the reef profile where reef communities did not begin until 12 m. Attempts were made to sample tissue and photograph every *Agaricia* spp*.* colony, however, owing to the abundance of small and cryptic (i.e. in covered locations) colonies, some colonies were likely missed, see electronic supplementary material, S1.1. Structure-from-motion photogrammetry was used to create three-dimensional (3D) point clouds by taking repeated photographs of the benthos and stitching these photographs together. Colony locations were manually annotated to the 3D point clouds by locating sampled colonies within the point cloud guided by video footage taken during sampling (detailed procedures in electronic supplementary material, S1.2).

### (b) Genotyping, taxonomic delimitation and clone identification

Extractions of genomic DNA were carried out using a salt extraction method [51] that has been optimized for corals. Single nucleotide polymorphism (SNP) markers were obtained using restriction site associated DNA sequencing (RADseq) based on a fusion of the GBS [52] and ddRAD methods [53] using *PstI* and *MspI* restriction enzymes; this method was designed by Hereward *et al.* [54] and is detailed in electronic supplementary material, S1.3. Six individual colonies of *A. lamarcki* and two of *A. grahamae* that were previously genotyped were supplied from Prata *et al.* [50] to identify the two previously discovered *A. lamarcki* taxa.

The full analysis pipeline, accompanying datasets, metadata and results can be found at GitHub [55]. Short read sequences were assembled (*de novo*) and variants were called using ipyrad v. 0.9.67 [56]; then, these were further filtered with VCFtools v. 0.1.16 [57]. Here, we focus solely on the cnidarian component of the holobiont and thus Symbiodiniaceae and other symbionts or contaminants were removed through blastn search comparing the *de novo* sequences to the *Cladocopium* spp. genome (e^−15^) and non-scleractinian matches on the NCBI nt-database (e^−4^). Heterozygosity and depth filtering provide a second layer of stringency for ensuring non-host sequences do not affect results. Individuals that had > 50% missing data were removed (203 individuals removed from an initial 970). Dataset filtering included a minimum read depth of five for each SNP, a maximum read depth three times greater than the average depth, minimum allele count of three and four missing data thresholds across SNPs (5, 10, 20 and 50%) for assessing consensus of genetic structuring results. Unsupervised and model-based clustering methods—principal component analysis (PCA) and Admixture v. 1.3.0 [58]—were used to detect distinct genetic clusters within all datasets: the proportion of variance and visual clustering (PCA), CV error and log-likelihood (Admixture) and biological relevance (PCA and Admixture) were all considered and compared to determine the nature of groupings (see electronic supplementary material, S1.4). After initial population structure analysis on a filtered dataset to confirm that identities based on morphology aligned with genetic group identity, individuals were assigned to the three taxonomic species (*A*. *agaricites*, *A*. *humilis* and *A*. *lamarcki*) and filtering and genetic structure analysis were repeated per species dataset.

To ensure that dispersal analyses focused on sets of individuals whose ability to reproduce was unimpeded by endogenous barriers to reproductive isolation, taxonomic species and taxa (putative cryptic species within taxonomic species) were delimited based upon Mallet’s ‘genotypic cluster’ definition [59]: *species* (applied to morphological descriptions of taxonomic species) and *taxa* (any group within the taxonomic species) are recognized if they form distinct genetic groups in sympatry and mostly contain individuals that are fully assigned to each group. When there is geographic differentiation, it is expected that sympatric taxa should be more differentiated than spatially structured populations (of the same taxon). Because these taxa are likely to be predominantly demographically independent, analyses were performed separately for each taxon. See electronic supplementary material, S1.4 and S2.1, which provide a greater explanation of how taxa were resolved.

For each species dataset, clones were identified using pairwise distances between individuals that included technical replicates (samples from different pieces of the same coral colony) to determine thresholds for sequencing error and somatic mutations. For all analyses (except clonal metrics), a single individual with the lowest missing data from each clonal group was retained. For non-clone datasets, outlier loci were identified with PCAdapt v. 4.3.3 [60] and removed and physical linkage was removed by randomly selecting one SNP per RAD contig.

### (c) Spatial analyses

Each photogrammetry plot was constructed in three dimensions with its own arbitrary local coordinate system. Coordinates for each plot were scaled and transformed for orientation (plots of different depths were aligned along the *y*/*z* axes and locations along the *x* axis). See electronic supplementary material, S1.5 for details on the preparation for spatial analyses and see electronic supplementary material, SF1 for a diagram of the plot configurations. Distances between centroids of colonies were used for spatial analyses.

Redundancy analysis (RDA) was used to assess the relative effects of spatial distance and depth distance on genetic variation, because both space and depth can independently contribute to genetic structure. Separate RDAs were run on each taxon using the *rda*() function in the R package vegan v. 2.6-2 [61], where individual genetic distance (Rousset’s *â*, [24]) were transformed in principal coordinates for the response matrix and the predictors were the coordinates *x*, *y* and *z* (depth) (see electronic supplementary material, S1.5.1).

Because RDA failed to show signals of within-taxon differentiation by depth, subsequent spatial analyses could be undertaken without considering depth. Thus, plots were oriented to the *xy* plane and distances were based only on 2D position (see electronic supplementary material, SF1). To obtain estimates of IbD slopes, we regressed Rousset’s *â* (genetic distance) and Loiselle’s *F* (genetic relatedness [62]) on the log of geographic distance between pairs of colonies ([25], eqn. 5).

(2.1) geneticmetric=a+b⋅ln(d)

These metrics were used because they scale linearly with log geographic distance under theoretical assumptions of IbD in 2D habitats. Regressions were restricted to distances within locations (e.g. distances ranging from 0.01 to 75 m) because these distances represented the sampled 2D area. Loiselle’s *F* is appropriate when there is inbreeding and/or selfing [28] and was used because some populations (grouped by location) had significantly positive *F*_*IS*_. Analyses using *â* and *F* were performed using different pipelines: for *â*, we used the R wrapper of GENEPOP v. 4.7.5 [63] and for *F*, we used SPAGeDi v. 1.5 [62]. For the SPAGeDi analyses, *σ* was iteratively estimated until the sampling range of distances spanned *σ* (minimum distance) to 10*σ*−50*σ* (maximum distance) (i.e. spatial scales shaped by recent drift and dispersal, [27,28]) using effective and census densities at appropriate neighbourhood area scales ([21]; see electronic supplementary material, S1.9). For GENEPOP analyses, approximate bootstrap confidence (ABC) intervals were used and for SPAGeDi, jackknifing across loci were used (see electronic supplementary material, S1.5.2). The regressions were deemed statistically significant when the 95% confidence intervals did not span 0. To capture larger spatial scales, we considered using 1D IbD analyses across locations [21,23]. However, significant population structure was detected within *A*. *agaricites* and *A*. *humilis* would confound IbD interpretation [64] and *A*. *lamarcki* taxa did not present IbD signals at this scale (see §3).

### (d) Kinship and sibship *N*_*e*_

Kinship analysis, performed with COLONY v. 2.0.6.8 [65], was conducted to confirm theoretical estimates of dispersal from IbD by assessing the distribution of kin distances as well as jointly estimating contemporary *N*_*e*_ for dispersal distance estimation. See electronic supplementary material, S1.6 for issues in uncertainty for estimating half-sibling relationships within COLONY. Populations were considered as having polygamous and monoecious mating systems with inbreeding and analyses were run per location when population structure was found among locations. Each analysis comprised 10 runs of medium-length, full-likelihood estimation with high precision. All individuals were considered as potential mothers, fathers and offspring because age cohorts were unknown (frequent partial mortality evidenced within *Agaricia* spp*.* impacts the ability to determine juvenile corals based on size [66]). If parent–offspring or sibling pairs (i.e. full-siblings or half-siblings) were found, spatial distances between them were calculated. Effective population size (*N*_*e*_) was estimated using the sibship method from Wang *et al*. [67] when siblings were present, and these estimates were divided by the spatial area covered by individuals in each location (including the areas between the depth replicated plots) to calculate *D*_*e*_. The sibship method for *N*_*e*_ is a contemporary estimate thus more likely related to the genetic neighbourhood when estimated within the neighbourhood area [67,68]. To ameliorate uncertainty in *N*_*e*_ estimates, we also present estimates based on census density (*N*_*c*_) from visual counts of the plots, which could be considered an upper bound on *N*_*e*_ as *N*_*c*_ is generally larger than *N*_*e*_ in invertebrates [69].

### (e) Neighbourhoods, densities and dispersal distances

A genetic neighbourhood [20] represents the effective number of individuals in a continuously distributed population that can easily mate or the effective number of individuals within a circle of radius 2*σ*, where *σ*^2^ is the mean squared axial distance between parent and offspring and *D* is the density or the number of the individuals per m^2^. Thus, the neighbourhood size (*NS*) for a 2D population is 4*πDσ*^2^. This value can be extracted as the inverse of an IbD slope (*b*) of genetic distance (*â*) and log geographic distance (based on eqn. 5 of Rousset [24]):


(2.2) $$4D\pi \sigma^{2}=\frac{1}{b}$$

Regarding Loiselle’s kinship (*F*), which decreases linearly with geographic distance and is sample dependent (i.e. metric is based on relatedness compared to the entire sample), an additional conversion is necessary to calculate the *NS*:


(2.3) $$\frac{-b}{\left(1-\;F_{\left(1\right)}\right)}=\frac{1}{4D\pi \sigma^{2}},$$

where *F*_*(1)*_ is the average relatedness among neighbours (i.e. the average relatedness at the first distance that includes all or most pairs of individuals) [28].

The dispersal variance (*σ^2^*) is not the variance in Euclidean dispersal distances but is the non-central second moment of the distribution of axial dispersal distances and thus can be used to characterize the distribution of dispersal distances if the distribution family is known. Given a Gaussian distribution of dispersal distances, *σ* is 1.25 times the mean Euclidean dispersal distance [70]. Extrapolating *σ* from neighbourhood size requires an independent estimate of population density (*D*). Two densities were calculated, one using census density (*D_c_* an approximation of the density of multilocus genotypes, see electronic supplementary material, S1.7) and the other using an effective density (*D_e_*) based on genetic effective population size (*N_e_*), derived from Wang’s sibship estimate (see above).

To characterize the uncertainty around each parameter (*NS*, *D*_*c*_ and *D*_*e*_), distributions were inferred using the central, upper and lower confidence intervals of both *NS* (derived from IbD slopes for *â* and *F* using equations (2.2) and (2.3)) and *N_e_* (from COLONYv2) and using each plot census estimate for *N_c_* see electronic supplementary material, S1.8 for further details where procedures were similar to Naaykens and D’Aloia; Pinsky *et al.* [29,30]. This random sampling from parameter distributions allowed the estimation of a probability distribution of dispersal distance (*σ*). Estimates of *σ* were calculated for instances when the slope of IbD (*b*) was statistically positive for genetic distance and negative for relatedness.

## 3. Results

### (a) Genotyping, taxonomic delineation and clone identification

Filtering yielded a dataset of 767 individuals and 15 659 SNPs across 919 loci (including the outgroup samples of 6 *A*. *lamarcki* and 2 *A*. *grahamae* from Prata *et al.* [50]). Population structure analyses of this ‘all individuals’ dataset found three highly divergent clusters, corresponding to the three taxonomic species: *A. agaricites*, *A. humilis* and *A. lamarcki* (see electronic supplementary material, S2.1). Subsequently, each species was processed independently and after clone removal and filtering (including outlier and physical linkage removal) yielded 335 individuals and 1606 SNP loci for *A. agaricites* (177 clones removed), 121 individuals and 1282 SNP loci for *A. humilis* (21 clones removed) and 92 individuals and 941 SNP loci for *A. lamarcki* (13 clones removed). Critical examination within each species found multiple taxa, where ‘taxa’ refers to putative cryptic taxa within taxonomic species and these terms will be used throughout (see electronic supplementary materials, S1.4 and S2.1). Within *A. agaricites,* we found two taxa (‘AA1’ and ‘AA2’); within *A. humilis*, we found three taxa (‘AH1’, ‘AH2’ and ‘AH3’) and within *A. lamarcki*, we found two taxa (‘AL1’ and ‘AL2’) (see electronic supplementary material, SF2−4). Some of these taxa differed in abundance by depth (see electronic supplementary material, table ST1). Specifically, AA1 was predominately found at 20 m depth, whereas AA2 was found at all depths sampled. AH2 and AH3 were restricted to 5 and 10 m whereas AH1 was found additionally at 20 m. For the taxa within *A. lamarcki* both were predominately found at 20 m with AL1 less abundant than AL2 overall. The *A. lamarcki* taxa were verified as being the same taxa previously identified by Prata *et al*. [50] using the outgroup samples (see electronic supplementary material, S2.1.2, SF8−9). We found further structuring within *A. agaricites* and *A. humilis* taxa related to geography (see electronic supplementary material, S2.1.2, SF4−7 and ST5). Each of these seven distinct taxa was analysed separately in the results that follow (see electronic supplementary material, ST1−2 for details of analysis datasets).

**Figure 2.**
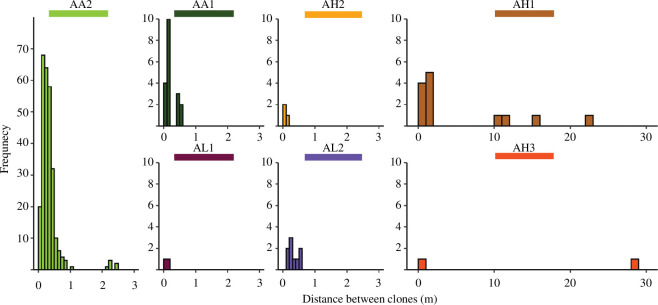
Finely distributed (across metres) spatial distances between clones in *Agaricia* taxa. For each taxon, the spatial distances between clones are on the *x*-axis and the frequency of pairs on the *y*-axis.

Across taxa, most of the clone groups comprised dyads (see electronic supplementary material, ST3 and SF11) and *A. agaricites* taxa had lower genotypic richness (i.e. less unique genotypes relative to total colonies, *Ng *: *N* of 0.6–0.7) compared to other taxa with *A*. *lamarcki* and *A*. *humilis* taxa exhibiting similar proportions (*Ng *: *N* of 0.8–0.9). Clonal spread within plots was spatially limited, with most clones under 1 m distant (figure 2). Four clone groups in two *A*. *humilis* taxa were between 10 and 30 m distant, and different clone group combinations were used to test the robustness of the IbD analyses (see electronic supplementary material, S1.5.3 and ST9).

### (b) Spatial analyses

Across all depth plots and locations, the RDAs showed that geographic distance in the *x*-coordinate predicted genetic distances within *A. agaricites* and *A. humilis* taxa (see electronic supplementary material, ST6) and that the explanatory contribution of depth (*z*-coordinate) was negligible. The *z*-coordinate was only marginally significant for AH1 (*p* < 0.04) and explained approximately 1% more of the variation than the *y*-coordinate thus both models including either *z* or *y* explained similar levels of variance. None of the explanatory variables explained significant variation within *A. lamarcki* taxa. Because depth was not found to structure variation within taxa, we only considered spatial distances for IbD results that follow.

**Figure 3.**
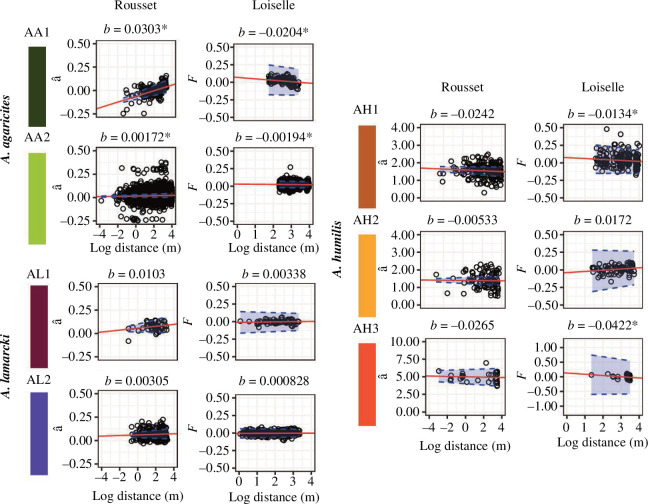
Isolation-by-distance (IbD) regressions used in dispersal estimation for *Agaricia taxa*. For each taxon, two regressions were made: one using Rousset’s genetic distance (*â*) and the other using Loiselle’s genetic relatedness (*F*)*,* with both metrics regressed across the log of geographic distance. For Rousset’s *â*, IbD is present for positive slopes (*b*) whereas for Loiselle’s *F* IbD is present for negative slopes (−*b*). For Loiselle’s *F* regressions, distances were truncated to be from *σ* to 20*σ* when *σ* could be estimated. Statistical significance of slopes was accepted by confidence intervals not spanning 0 (or for AA2 when *p* < 0.05 using Rousset’s *â*). ‘*’ signifies statistical significance.

Taxa within species exhibited varied patterns of IbD among genetic metrics (figure 3; see elecronic supplementary material, ST7−8). For *A. agaricites* taxa, IbD patterns were consistently significant. For *A. humilis* taxa, which were highly inbred (see electronic supplementary material, ST5; *F_IS_* = 0.5–0.8), Loiselle’s kinship metric was the most appropriate measure and showed significant IbD patterns that were not found when using Rousset’s *â*, except for the AH2 taxon. Likewise for *A. lamarcki* taxa, which showed different patterns among the two metrics and also had inbreeding (see electronic supplementary material, ST5; *F_IS_* = 0.04–0.05), the IbD slopes were not apparent using *F* but were positive (not significant) when using Rousset’s *â*. These positive slopes within *A. lamarcki* taxa using *â* are likely spurious because of inbreeding, the lack of population structure among locations (see electronic supplementary material, ST5) and the lack of spatial signals in the RDA results (see electronic supplementary material, ST6). *A. agaricites* and *A. humilis* had significant population structure across larger distances (see electronic supplementary material, ST5) consistent with finding IbD across smaller spatial scales.

### (c) Kinship and sibship *N_e_*

Kin were identified for both *A. agaricites* taxa and *A. humilis* taxa within some locations; however, no kin were found for *A. lamarcki* taxa (see electronic supplementary material, ST10), matching the lack of spatial structure signals. Three parent-offspring pairs were found for *A. agaricites*, two of AA1 and one of AA2 and all three pairs were found within a few metres (< 3 m; see electronic supplementary material, SF13). Because siblings were present, *N_e_* was calculated using the sibship method for both *A. agaricites* taxa and *A. humilis* taxa (see electronic supplementary material, ST10) and were used for effective density measures in *σ* estimation. Low numbers of kin occurred in the *Agaricia* samples, impacting the ability to determine true relationships of half-siblings, which comprised of most of the kin found in all taxa and with half-siblings being the only kin found within *A. humilis*. Thus, the *N_e_* estimates are approximate (see electronic supplementary material, S1.6 and §4).

### (d) Neighbourhoods, densities and dispersal distances

Distributions of genetic neighbourhood size were calculated from the IbD slopes and were combined with both distributions of census (*D*_*c*_) and effective (*D*_*e*_) densities to create probability distributions of *σ*. For both *A*. *agaricites* taxa and two *A*. *humilis* taxa, the median *σ* estimates were across metres (2–11 m; table 1; see electronic supplementary material, SF14) using Loiselle’s *F* as the genetic metric (both genetic metrics for AA1, â and *F*) and regardless of density estimator (*D*_*c*_ and *D*_*e*_). Furthermore, for these *σ* estimates, IbD was measured at the ideal spatial scale (namely from a distance range of *σ* as the minimum distance used to 10*σ*−50*σ* as the maximum distance used) with densities estimated at the ideal area (i.e. the neighbourhood area, 4π*σ*^2^) and population structure analyses were consistent with this result (positive *F*_*ST*_ among locations; see electronic supplementary material, ST5, S1.9 and ST12). Estimating *σ* using â for AA2 generated a large probability distribution of estimates and a much larger central *σ* estimate, this was owing to the confidence interval for the slope spanning 0 and thus giving an infinite upper confidence interval for neighbourhood size. For AA2, the *σ* estimate using *F* is likely more reliable owing to some inbreeding occurring within AA2 populations. For AH2 and the two *A*. *lamarcki* taxa (using *F*), *σ* could not be estimated (slopes were inconsistent with IbD expectations; figure 3).

**Table 1.** Generational dispersal distances were found to be within metres for *Agaricia agaricites* (AA1 and AA2) and *A. humilis* (AH1 and AH3) taxa. For each taxon IbD analyses were performed at the ‘within location’ scale (where samples were distributed in 2D) with the maximum distance scales and the number of locations and plots used in all analyses shown. Individual densities comprised of both census (*D*_*c*_) from raw counts and genetic effective population size (*D*_*e*_) estimated from sibship method in COLONY v. 2. Neighbourhoods (NS) were extracted from IbD slopes using Rousset’s *â* and Loiselle’s *F* versus the log of geographic distances and when combined with density estimates gave estimates of dispersal distance (*σ*). Using the uncertainty around all estimates, distributions were created, resampled from and combined to yield probability distributions for *σ*. The median estimate of *σ* is shown from redrawing values of 2000 simulations for each metric (*D*_*c*_, *D*_*e*_ and NS) with the 90% probability intervals shown in brackets.

spatial scales and plot replicates			census and effective population density		neighbourhood and *σ* based on Rousset’s *â*			neighbourhood and *σ* based on Loiselle’s *F*		
taxon	scale (m)	loc (plots)	*D*_*c*_ (m^2^)	*D*_*e*_ (m^2^)^a^	NS	*σ*_*c*_ (m)	*σ*_*e*_ (m)	NS	*σ*_*c*_ (m)	*σ*_*e*_ (m)
AA1	30	4 (6)	0.26	0.11	35	3.17	5.12	44	3.64	5.91
			(0.14–0.62)	(0.07–0.17)	(4–2309)	(1.05–25.8)	(1.79–41.31)	(4–6624)	(1.01–44.46)	(1.73–72.96)
AA2	75	4 (11)	0.89	0.38	758114	256.01	399.79	587	7.39	11.04
			(0.58–1.39)	(0.2–0.81)	(9–7.3e18)^b^	(0.88–7.1e8)^b^	(1.29–1.1e9)^b^	(13–8.1e6)	(1.05–874.87)	(1.57–1337.06)
AH1	55	4 (8)	0.33	0.24	—^c^	—^c^	—^c^	94	4.77	5.67
			(0.22–0.55)	(0.13–0.39)				(9–9202)	(1.49–44.3)	(1.73–53.59)
AH3	33	3 (4)	0.22	0.39	–^c^	—^c^	—^c^	23	2.75	2.21
			(0.15–0.57)	(0.17–0.82)				(7– 197)	(1.31–8.27)	(1.04–6.96)

^a^ Location and plot replicates in table do not apply for *N_e_* estimates for AA1, AH1 and AH3 because only one location estimate for each taxon was used.

^b^ Upper confidence intervals approach infinity due to a negative slope upper confidence interval in the regression, which was truncated at 0 for *σ* estimates. Loiselle’s estimate is more appropriate due to detected inbreeding.

^c^ Measures were not extracted due to absence of a significant IbD slope.

## 4. Discussion

In this comparative study of dispersal estimates in *Agaricia* corals, we found generational dispersal distances between 2 and 11 m for four of the seven taxa studied (two *A. agaricites* and two *A. humilis* taxa; table 1). These estimates are within the ideal spatial distance range for dispersal estimation (namely where migration-drift equilibrium is met: a spatial range of *σ* to 10*σ*–50*σ* [27,28]), with effective density measured at the scale of the neighbourhood area (namely areas of mostly random mating: 4π*σ*^2^ [21,68]). In contrast, the two *A. lamarcki* taxa did not present an IbD signal and thus we could not estimate dispersal distances but infer from their lack of spatial genetic signals across the length of study that their dispersal is likely much greater than the other taxa considered. This contrast among taxa, which are often assumed to be ecologically equivalent [41], indicates the potential for major differences in their life history strategies. Across *Agaricia* taxa, asexual dispersal is unlikely to have much impact on gene dispersal as most clones were near each other (approx. 1–2 m; figure 2). Thus, populations of *A. agaricites* and *A. humilis* are predominantly locally retained owing to their short dispersal distances, making them more susceptible to local extinction after large-scale disturbances but also more likely to repopulate if proximate populations persist. Depth did not appear to influence dispersal for any taxon. This study demonstrates that structure-from-motion photogrammetry combined with genomic-level genotyping can support spatially explicit genetic studies for benthic organisms and enable comparisons across multiple species and locations.

### (a) Implications of short dispersal

All *A. agaricites* and *A. humilis* taxa exhibited spatial genetic structure among locations (see electronic supplementary material, ST5) and IbD correlations within locations (except AH2; table 1 and figure 3). Other studies on brooding corals have also found spatial genetic patterns consistent with restricted dispersal. For example, IbD correlations within brooders were found across metres [39,71,72], whereas broadcasters lacked spatial patterns across 10s of km [73,74]. Our estimates of *σ* are equivalent to IbD-derived *σ* estimates found within brooding *Pocillopora damicornis* (2.9–3.8 m [34]), larger than those of *Corallium rubrum* (0.15–0.21 m [31]) and much smaller than that of broadcaster *Acropora palmata* (1 km [35]). Additionally, *A. agaricites* and *A. humilis* taxa dispersed smaller but similar distances to the broadcasting fire coral, *Millepora cf. platyphylla*, which has low mobility propagules where direct observation indicated that 65% of the offspring observed settled within 300 m of parents [15]. Whether the short dispersal distances found in our study and for *P. damicornis* represent general dispersal patterns for brooding scleractinians is unresolved but represents an interesting consistency.

Generational dispersal distances of 2–11 m suggest that most ecologically significant dispersal happens at the scale of metres. Large-scale disturbances that cause extensive mortality could, therefore, cause local extinctions for these taxa. On the other hand, remnant patches of restricted dispersal taxa retain offspring close and could repopulate a local area. While generational dispersal distance (*σ*) gives us a good idea of the most frequent range of dispersal distance, it does not describe the shape of the dispersal kernel. If dispersal kernel tails are long (i.e. rare events of long-distance dispersed propagules) as expected in marine populations [75], then rare long-distance dispersal events combined with frequent short-distance dispersal may allow these taxa to be good colonizers [76]. This strategy may have led to the large species ranges of *A*. *agaricites* and *A*. *humilis* across the Caribbean.

Local adaptation should scale inversely with increasing dispersal [77]. Thus, local adaptation to certain habitats across fine scales would be better facilitated by limited dispersal (e.g. varying thermal tolerances within lagoon versus crest versus slope). Paradoxically, then, notable examples of fine-scale local adaptation in corals come from broadcast spawners [78–80]. If brooders also show adaptation to specific microhabitats, then local reef populations may shelter considerable standing genetic variation that might aid rapid evolutionary change such as to climate change stress. Dispersal is often assumed to be large for corals, but if dispersal distances are limited for some species of coral (especially brooders), then the dynamics of local effects (e.g. local replenishment and adaptation) may be more important than previously assumed.

### (b) Variance in dispersal among congeners

Within locations, *A. agaricites* (AA1 and AA2) and *A. humilis* (AH1 and AH3) exhibited metre-scale dispersal. These taxa also showed evidence of population structure between sampling locations (see electronic supplementary material, SF4, S5 and S7 and ST5). In contrast, results for *A. lamarcki* were inconsistent, showing positive (but non-significant) IbD slopes for Rousset’s metric but not having the expected negative slopes for Loiselle’s metric (figure 3). Loiselle’s metric is likely more reliable given the inbreeding present within these taxa (see electronic supplementary material, ST5) [28,68]. In addition, *A. lamarcki* taxa presented no spatial patterns (see electronic supplementary material, ST5,6), implying that sampling sites were insufficiently distant to capture IbD. IbD analyses across larger spatial scales for *A. lamarcki* taxa may be more appropriate if dispersal is larger (compared to AA1, AA2, AH1 and AH3). Genetic homogeneity in *A. lamarcki* across a 43 km distance matches previous results for similar spatial scales across both shallow and mesophotic depths [50].

Demographic parameters, including dispersal, census and effective population sizes, are laborious to obtain, and it is tempting to substitute values for congeners with the implicit assumption that traits are evolutionarily conserved. We find, however, that dispersal may differ greatly amongst *Agaricia* species. This possible contrast in dispersal among *A*. *agaricites* and *A*. *humilis* taxa versus *A*. *lamarcki* taxa corresponds to the deep evolutionary divergence of two clades within the genus between *A*. *agaricites*/*A*. *humilis*/*A*. *tenuifolia* and *A*. *lamarcki*/*A*. *undata*/*A*. *grahamae*/*A*. *fragilis* [81]. The reproductive mode of *A*. *lamarcki* has not been assessed, nor has it for *A*. *undata*, *A*. *grahamae* and *A*. *fragilis*; thus, it is possible these species have broadcasting reproductive modes. Congeners may differ in dispersal propensities and thus it is important to assess each species individually.

### (c) Clonal propagation at the finest scale

Asexual reproduction within *Agaricia* corals was evident at the finest scale (approx. 1 m; figure 2), especially within *A. agaricites* taxa, although there was some evidence for longer asexual dispersal within *A. humilis* taxa (10–30 m, figure 2). Finely distributed clones probably result from fission, where one colony separates into multiple smaller colonies due to partial mortality, as observed by earlier works on *Agaricia* corals [66,82]. However, the larger distances between clones found in *A. humilis* taxa could be due to the dispersal of parthenogenic larvae. Despite the moderately low ratios of unique genotypes to total colonies for *A. agaricites* taxa (*Ng *: *N* = 0.6–0.7), the number of clone groups per genotype was low (see electronic supplementary material, SF11) and the number of genetically distinct colonies within close proximity to each other was high (see electronic supplementary material, SF12). Overall, the spread of genetic variation is predominantly shaped by sexual reproduction.

### (d) Estimating generational dispersal distances across more coral species

Dispersal distance estimates for scleractinian corals are urgently needed to document the variation in dispersal across different functional groups and geographic regions. This study demonstrates that individual-based IbD methods can be used to gain insights into coral dispersal. Studies using IbD are increasingly showcasing that dispersal distance for many marine fishes is smaller than anticipated from traditional population genetic studies [30,83], and it seems likely that similar results could be found for corals, as shown in the present study and other examples (e.g. [31,34,35]).

A novel aspect of the present study is the use of photogrammetry to map colonies and measure census densities in 3D. Our methods were more labour-intensive than strictly necessary as these data are supporting multiple purposes and projects [40]. Photogrammetric mapping can be inexpensive (using simple action cameras), fast (covering several hundred square metres in a single dive) and relatively straightforward in terms of processing protocols. The advantages of advanced 3D-photogrammetric mapping implemented here include incorporating counts of organisms found in cryptic locations (i.e. under overhangs or in crevices) and accurately determining their spatial positions, which may not be accounted for in traditional surveys of structurally complex reefs. Overall, combining photogrammetry with individual-based genotyping can be an incredibly useful design to estimate dispersal distances for corals and other benthic organisms using IbD approaches.

Best practice for estimating dispersal from IbD involves iterative cycles of analysis by adjusting the spatial scales of the IbD regression (between distances from lower bound *σ* to an upper bound in the range of 10*σ*−50*σ*) [27,28], adjusting the spatial area of effective density to the neighbourhood scale (4π*σ*^2^) [21,68] and re-estimating *σ* following criteria 1 and 2 until all estimates are consistent with each other and consistent with independent analyses (e.g. spatial population genetic structure found from assignment methods or *F*_*ST*_ estimates). Given that this process starts within an unknown *σ* and that dispersal could be extensive, ideal sampling will include varying distances such as by using intensive sampling at multiple reefs or using transects to create continuous distance classes. For example, if we had included more samples at intermediate spatial distances (i.e. between 100 m and 10 km) and larger distances (> 43 km), more spatial scales could be queried with some of these scales perhaps better within the migration-drift equilibrium and thus improving *σ* accuracy for some taxa (see electronic supplementary material, S1.9 and ST12) and resolving *σ* for the *A*. *humilis* taxon (AH2) and *A*. *lamarcki* taxa. Estimating contemporary *N*_*e*_ has long been problematic. However, both sibship [67] and linkage disequilibrium [84] methods show great promise if sufficient sample sizes and appropriate samples are used [68,84,85]. Sibship *N*_*e*_ methods are best suited to populations with localized dispersal and sampling techniques that target exhaustive sampling of an area. Although our sibship *N*_*e*_ estimates are uncertain owing to the low numbers of kin found, we found highly similar *N*_*c*_ estimates. These taxa, owing to their short dispersal, could have similar *N*_*c*_ and *N*_*e*_. Regardless, including *N*_*c*_ provides independent benchmarking for *N*_*e*_ if there are concerns about methodological bias (see electronic supplementary material, S1.6). Given that the most probable *σ* estimates were of a few metres, the areas where we sampled were equivalent to the estimated neighbourhood areas and thus the magnitude of our density estimates is likely of an appropriate scale. Indeed, we believe the most difficult parts of these analyses are choosing the appropriate spatial scales for *N*_*e*_ and IbD slope estimates. Advances in technology (i.e. large genomic datasets and photogrammetric mapping) improve the accuracy and efficiency of querying spatial and genetic relationships, allowing us to fast-track our understanding of genetic neighbourhoods and dispersal in benthic organisms.

## 5. Conclusions

Quantifying coral dispersal distances is important, given the rapidly declining coral populations worldwide. Spatially explicit population genomic studies of benthic coral reef organisms enable joint estimation of density and IbD to obtain generational dispersal distances. We found extremely short generational dispersal distances within several *Agaricia* taxa, indicating that demographic replenishment is unlikely from reefs kilometres apart; however, local support is likely to sustain populations. Maintaining demographic stability and connectivity is key for population recovery over ecological timescales, and dispersal enables gene flow to distribute the genetic variation needed to withstand environmental changes. Generational dispersal distances should be measured for more coral species because it is a critical parameter for predicting recovery and adaptation and thus for guiding the management of threatened populations and species.

## Data Availability

Raw genomic data is deposited on NCBI Sequence Read Archive (SRA) BioProject Accession PRJNA1120949 and linked metadata deposited on the GEnomic Observatories MetaDatabase (GEOME) [86]. All code and derived data for analyses, tables and figures is deposited on GitHub [55] and Zenodo [87]. Supplementary material is available online [88].
